# Regulation of Proinflammatory Molecules and Tissue Factor by SARS-CoV-2 Spike Protein in Human Placental Cells: Implications for SARS-CoV-2 Pathogenesis in Pregnant Women

**DOI:** 10.3389/fimmu.2022.876555

**Published:** 2022-04-07

**Authors:** Xiaofang Guo, Nihan Semerci, Viviana De Assis, Umit A. Kayisli, Frederick Schatz, Thora S. Steffensen, Ozlem Guzeloglu-Kayisli, Charles J. Lockwood

**Affiliations:** ^1^Department of Obstetrics and Gynecology, University of South Florida, Morsani College of Medicine, Tampa, FL, United States; ^2^Department of Pathology, Tampa General Hospital, Tampa, FL, United States

**Keywords:** SARS-CoV-2, ACE2, TMPRSS2, proinflammatory cytokines, tissue factor, trophoblast, pregnancy

## Abstract

SARS-CoV-2 infects cells *via* binding to ACE2 and TMPRSS2, which allows the virus to fuse with host cells. The viral RNA is detected in the placenta of SARS-CoV-2-infected pregnant women and infection is associated with adverse pregnancy complications. Therefore, we hypothesize that SARS-CoV-2 infection of placental cells induces pro-inflammatory cytokine release to contribute to placental dysfunction and impaired pregnancy outcomes. First, expression of *ACE2* and *TMPRSS2* was measured by qPCR in human primary cultured term cytotrophoblasts (CTBs), syncytiotrophoblast (STBs), term and first trimester decidual cells (TDCs and FTDCs, respectively), endometrial stromal cells (HESCs) as well as trophoblast cell lines HTR8, JEG3, placental microvascular endothelial cells (PMVECs) and endometrial endothelial cells (HEECs). Later, cultured HTR8, JEG3, PMVECs and HEECs were treated with 10, 100, 1000 ng/ml of recombinant (rh-) SARS-CoV-2 S-protein ± 10 ng/ml rh-IFNγ. Pro-inflammatory cytokines *IL*-*1β*, *6* and *8*, chemokines *CCL2*, *CCL5*, *CXCL9* and *CXCL10* as well as tissue factor (*F3*), the primary initiator of the extrinsic coagulation cascade, were measured by qPCR as well as secreted IL-6 and IL-8 levels were measured by ELISA. Immunohistochemical staining for SARS-CoV-2 spike protein was performed in placental specimens from SARS-CoV-2–positive and normal pregnancies. *ACE2* levels were significantly higher in CTBs and STBs *vs.* TDCs, FTDCs and HESCs, while *TMPRSS2* levels were not detected in TDCs, FTDCs and HESCs. HTR8 and JEG3 express *ACE2* and *TMPRSS2*, while PMVECs and HEECs express only *ACE2*, but not *TMPRSS2.* rh-S-protein increased proinflammatory cytokines and chemokines levels in both trophoblast and endothelial cells, whereas rh-S-protein only elevated *F3* levels in endothelial cells. rh-IFNγ ± rh-S-protein augments expression of cytokines and chemokines in trophoblast and endothelial cells. Elevated *F3* expression by rh-IFNγ ± S-protein was observed only in PMVECs. In placental specimens from SARS-CoV-2-infected mothers, endothelial cells displayed higher immunoreactivity against spike protein. These findings indicated that SARS-CoV-2 infection in placental cells: 1) induces pro-inflammatory cytokine and chemokine release, which may contribute to the cytokine storm observed in severely infected pregnant women and related placental dysfunction; and 2) elevates *F3* expression that may trigger systemic or placental thrombosis.

## Introduction

Coronaviruses (CoV) family members are large, enveloped, single-stranded, and positive sense RNA viruses that present in many avian and mammalian species. In humans, CoV usually causes mild to moderate upper-respiratory tract illnesses. However, Severe Acute Respiratory Syndrome (SARS)-CoV and Middle East Respiratory Syndrome (MERS)-CoV can have fatal outcomes (1, 2). A novel SARS-like CoV, SARS-CoV-2, was identified in Wuhan, China, in December 2019, and rapidly spread and mutated (3–5), producing the current prolonged pandemic of coronavirus disease 2019 (COVID-19) (6). The SARS-CoV-2 virus primarily infects the respiratory tract (7), and infected patients exhibit a wide range of symptoms from mild to severe respiratory distress (1, 8). However, SARS-CoV-2 infection can result in additional disease-associated symptoms in different organ systems such as poor appetite, nausea, vomiting, and diarrhea in the digestive system, headache and confusion in the nervous system, and chest distress and cardiac injury in the cardiovascular system (1, 8).

SARS-CoV-2 infects host cells that express angiotensin-converting enzyme 2 (ACE2) mainly located in lung, heart, ileum, and kidney (9). The initial step of viral entry is mediated by spike (S) protein on the viral surface. The S-protein binds to its ACE2 receptor *via* its receptor-binding domain (9), and is proteolytically activated by type II transmembrane serine protease (TMPRSS2), present on the surface of the host cell (10). S-proteins activation leads to conformational changes that allow viral entry, release the viral RNA into cytoplasm to generate new viral particles (11). SARS-CoV-2 entry into host cells is a crucial factor for viral permissiveness and pathogenesis.

Pregnant women represent a vulnerable population for COVID-19 infection. Moreover, SARS-CoV-2 infection in pregnant women results in more severe symptoms compared with non-pregnant women, although pregnancy does not increase susceptibility to infection (12, 13). Recent studies reported that SARS-CoV-2 infected pregnant women are more likely to be hospitalized with increased risk for intensive care unit admission and higher mortality rates *versus* infected non-pregnant women (12, 14, 15). Furthermore, SARS-CoV-2 infection increases rates of cesarean delivery and preterm birth (PTB) and/or other pregnancy outcomes including low birth weight, stillbirth, abruption, and preeclampsia (13, 16, 17), especially when women are infected in the third trimester (18, 19). These complications may be related to the unique adaptation of the maternal immune system at different stages of pregnancy: a pro-inflammatory state that enhances implantation and the initiation of labor in the 1^st^ and 3^rd^ trimesters, respectively and an anti-inflammatory state facilitating fetal growth in the 2^nd^ trimester (20). Although, vertical transmission of SARS-CoV-2 remains highly debated, viral RNA is detected in placental villi, predominantly in syncytiotrophoblasts, cytotrophoblasts, villous fibroblasts, Hoffbauer cells, and endothelial cells (21, 22).

Therefore, we hypothesized that SARS-CoV-2 induces utero-placental pro-inflammatory cytokine and chemokine release, as well as activation of the coagulation cascade, consistent with the cytokine storm and prothrombotic state associated with severe infections. This, in turn, places pregnant women and their fetuses at higher risk for severe complications. Thus, we initially compared expression levels of potential viral entry receptors in maternal (decidual), fetal (trophoblastic), and endothelial cell cultures, and then explored the expression levels of pro-inflammatory cytokines, chemokines, and coagulation factor III (*F3*; *aka* tissue factor) in recombinant (rh-) spike (S)-protein of SARS-CoV-2-treated trophoblast and endothelial cell cultures.

## Materials and Methods

### Cell Culture and Recombinant Proteins

This study was approved by the University of South Florida Institutional Review Boards (Pro00019480). Human first trimester immortalized extravillous trophoblast cells (HTR8/SV^neo^) and choriocarcinoma trophoblast cells (JEG3) (ATCC, Manassas, VA) were cultured in phenol-free basal medium (DMEM/F12, Thermo Fisher Scientific, Waltham, MA) with 10% fetal bovine serum (Thermo Fisher Scientific) and 1% antibiotic-antimycotic complex (Gibco, Thermo Fisher Scientific). We chose human placental microvascular endothelial cell (PMVEC), which is an excellent *in vitro* model to study vascularization in the placenta (23), to evaluate fetal microvascular endothelial responses against SARS-CoV-2 S-protein, whereas human endometrial endothelial cells (HEECs) were chosen to evaluate maternal microvascular endothelial responses against SARS-CoV-2 S-protein. Frozen PMVECs is a kindly gift from Dr. Hana Totary-Jain (USF), purchased from ScienCell Research Laboratories (Carlsbad, CA). According to the manufacturer’s instruction, PMVECs are obtained from healthy pregnant women and characterized by immunofluorescence with antibodies specific to vWF/Factor VIII. As characterized previously (24), frozen HEECs were isolated from endometrial biopsies obtained from healthy women, who were not under hormonal treatment. Frozen PMVECs and HEECs were thawed and cultured in EGM-2 medium supplemented with low serum growth supplement (Gibco, Thermo Fisher Scientific) with 1% antibiotic-antimycotic complex. SARS-CoV-2 rh-S-protein was provided by BEI Resources (Manassas, VA). Human recombinant interferon gamma (rh-IFNγ) was purchased from R&D systems (Minneapolis, MN).

### Experimental Design

Confluent HTR8, JEG3, HEECs and PMVECs cultures were trypsinized and seeded in 6-well culture plates (1×10^5^ cells/well). The next day, the cells were exposed to either mock (control) or rh-S-protein at concentrations of 10, 100 and 1000 ng/ml, or 10ng/ml rh-IFNγ ± 10 ng/ml rh-S-protein in 500 µl serum free media and then shaken every 15 min to enhance rh-S-protein binding to cells at 37°C for 1 hour. Thereafter, 1500 µl fresh media with serum was added into cells. After 24 hours, plates were washed 3 times with ice-cold PBS and stored at −80°C for further RNA extraction.

### RNA Isolation, Reverse Transcription, and qPCR

Total RNAs from HTR8, JEG3, PMVECs and HEECs cultures were isolated using RNeasy Mini Kit (Qiagen, Germantown, MD) followed by DNase I treatment (Qiagen) to eliminate genomic DNA contamination. To compare endogenous expression of SARS-CoV-2 entry molecules, *ACE2* and *TMPRSS2* in fetal and maternal cells, previously isolated RNAs from primary cultured term cytotrophoblasts, syncytiotrophoblasts, term decidual cells, first trimester decidual cells and human endometrial stromal cells were employed (25). Reverse transcription using RETROscript kit (Ambion, Austin, TX) was performed as described (26) and qPCR performed using TaqMan gene expression assays to detect gene expression levels of: 1) pro-inflammatory cytokines interleukin (*IL)-1β*, *IL-6*, and *IL-8*; 2) chemokines C-C motif chemokine ligand (*CCL*)*2 -5* as well as C-X-C motif chemokine ligand (*CXCL*) *9* and *10*; and 3) tissue factor (*F3*) (Applied Biosystems, Grand Island, NY, TaqMan assay ID given in **Supplementary Table 1**). All reactions were performed in duplicate. Expression of the target genes was normalized to β-actin levels, and the 2^−ΔΔCT^ method was used to calculate relative expression levels (27).

### Enzyme-Linked Immunosorbent Assay

Media from HTR8 and PMVEC cultures treated with vehicle 10 or 1000 ng/ml rh-S-protein or 10 ng/ml IFNΥ ± 10 ng/ml rh-S-protein for 24 hours were collected, centrifuged and the resultant supernatants were stored at -80^0^C. Secreted IL-6 and IL-8 levels were measured using specific enzyme-linked immunosorbent assay (ELISA) kits (R&D Systems; Minneapolis, MN). Briefly, 96-well ELISA microplates were coated with a capture antibody; after blocking with 5% BSA, 1:4 diluted samples were added to the coated plates for 2 h, followed by a biotin-conjugated detection antibody. Antibody binding was measured with horseradish peroxidase-conjugated avidin along with a soluble colorimetric substrate. The absorbance was read at 450 nm using a microplate reader (Bio-Rad). Each sample was measured in duplicate

### Immunohistochemical Staining

After receiving IRB approval, placental specimens from SARS-Cov-2 infected mothers (n=3) who tested positive for COVID-19 in the third trimester and gestational age-matched normal pregnancies (n=3) were obtained from Clinical Pathology Laboratories at Tampa General Hospital. 5 μm formalin-fixed paraffin embedded placental sections were processed for immunohistochemistry as described previously (28). Briefly, after deparaffinization and rehydration, paraffin-embedded sections were boiled in 10 mM citric acid solution (pH: 6.0) for antigen retrieval for 20 min and incubated in 3% H_2_O_2_ for endogenous peroxidase quenching for 10 min. The slides were incubated with 10% goat serum (Vector Labs, Burlingame, CA) for 30 min at room temperature, then overnight with mouse monoclonal anti-SARS-Cov-2 Spike RBD (monoclonal mouse IgG2A, clone no. 1035423, 10µg/ml dilution; R&D Systems, Minneapolis, MN). For negative control, placental sections were incubated with non-immune mouse IgG_2a_ in place of primary antibody at the same concentration. All sections were washed in PBS containing 0.1% Tween-20 (PBS-T) and incubated with biotinylated anti-mouse IgG antibody (1/400 dilution; Vector Labs) for 30 min. Following several rinses in PBS-T, the sections were incubated in streptavidin–peroxidase complex (Elite ABC Kit, Vector Labs) for 30 min. After washing, slides were exposed to diaminobenzidine tetrahydrochloride dehydrate (Vector Labs) as a chromogen for 3 min and counterstained with hematoxylin before permanent mounting.

### Statistical Analysis

Results were analyzed by One-Way ANOVA followed with a *post-hoc* Tukey test if normally distributed or using the Kruskal-Wallis test followed by the *post-hoc* Student-Newman-Keuls’ test if non-parametrically distributed using SigmaStat version 3.0 software (Systat Software, San Jose, CA), *P<0.05* was considered statistically significant.

## Results

### Expression of *ACE2* and *TMPRSS2* Viral Entry Molecules in Fetal and Maternal Cells

**Figure 1:**
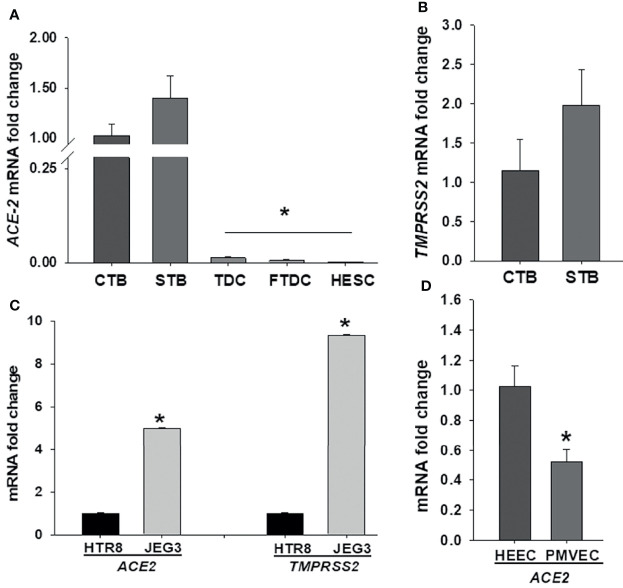
Comparison of SARS-CoV-2 entry receptor *ACE2* and *TMPRSS2* mRNA levels among various decidual (maternal) and placental (fetal) cell types at the maternal-fetal interface. Expression levels of *ACE2* **(A)** and *TMPRSS2* **(B)** in human term cytotrophoblast (CTBs), syncytiotrophoblast (STBs), term decidual cell (TDCs), first trimester decidual cell (FTDCs), and human endometrial stromal cell (HESCs) cultures; Expression levels of *ACE2* and *TMPRSS2* in trophoblastic cell lines HTR8 and JEG3 **(C)**; as well as expression levels of *ACE2* in placental microvascular endothelial (PMVECs) and human endometrial endothelial cell (HEECs) cultures **(D)** by qPCR. Bars represent mean ± SEM, n=4; * *P<0.05 vs.* CTB or STB **(A)**; * *P<0.05 vs.* HTR8 **(C)**; * *P<0.05 vs.* HEECs **(D)**.

To elucidate SARS-CoV-2 cell tropism in the placenta, we first investigated the expression levels of the cell entry receptor, *ACE2*, and priming protease, *TMPRSS2* at the maternal-fetal interface including primary cultured term cytotrophoblasts, syncytiotrophoblasts, term decidual cells, and first trimester decidual cells as well as human endometrial stromal cells obtained from non-pregnant women. qPCR analysis revealed that *ACE2* mRNA levels are significantly higher in both cytotrophoblasts and syncytiotrophoblasts (Ct < 30) *vs.* term or first trimester decidual cells as well as endometrial stromal cells (Ct > 33) (**Figure 1A**). While both trophoblastic cells displayed weak *TMPRSS2* expression (Ct > 33), maternal stromal decidual cells did not express *TMPRSS2* (**Figure 1B**). Subsequently, we compared *ACE2* and *TMPRSS2* levels in trophoblastic cell lines HTR8, and JEG3, and detected significantly higher *ACE2* (~ 4.9-fold) and *TMPRSS2* (~9-fold) levels in JEG3 than HTR8 cells (**Figure 1C**). In addition to trophoblast cells, we also compared *ACE2* and *TMPRSS2* levels in fetal and maternal endothelial cell types PMVECs from placental specimens and HEECs, respectively to explain COVID-19 severity in pregnancy. qPCR results revealed weak *ACE2* mRNA levels in both cell types and a slightly higher in HEECs (Mean ± SEM; 1.02 ± 0.13) compared to PMVECs (0.52 ± 0.08) (**Figure 1D**). In contrast, *TMPRSS2* levels were undetectable in both endothelial cell types.

### Increased Inflammatory Cytokine Expression Induced by rh-S-protein in Trophoblast and Endothelial Cells

**Figure 2:**
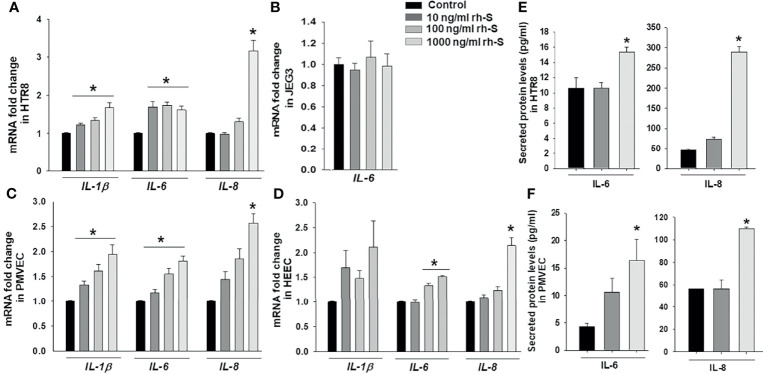
Induction of pro-inflammatory cytokine in trophoblast and endothelial cell cultures by rh-S-protein. SARS-CoV-2 induced expression of pro-inflammatory cytokines *IL-1β*, *IL-6* and *IL-8* mRNA levels in trophoblastic cells HTR8/SV^Neo^ **(A)** and JEG3 **(B)** as well as PMVEC **(C)** and HEEC cultures **(D)** treated with 10, 100, or 1000 ng/ml rh-S-protein *vs*. mock-treated control treatment. Bars represent mean ± SEM, n=4; * *P<0.05 vs.* control; and *IL-8* mRNA * *P<0.05 vs.* control or 10 or 1000 ng/ml rh-S protein **(A, C, D)**. Secreted IL-6 and IL-8 protein levels in culture supernatants of HTR8 **(E)** and PMVECs **(F)** treated with control or 10 or 1000 ng/ml rh-S-protein. Bars represent mean ± SEM, n=3; * *P<0.05 vs.* control **(E, F)**.

To mimic inflammatory changes induced by SARS-CoV-2 in the placenta, and to explore virus-induced pregnancy outcomes, HTR8 and JEG3 cell lines as well as PMVECs and HEECs were treated with 10, 100, 1000 ng/ml rh-S-protein for 24 hours and the expression of pro-inflammatory cytokine genes *IL-1β*, *IL-6*, and *IL-8* measured by qPCR. In HTR8 cells, rh-S-protein treatment significantly increased levels of *IL-1β* and *IL-6 vs.* controls, displaying a clear dose-response effect to increasing concentrations of rh-S-protein, whereas only 1000 ng/ml rh-S-protein significantly induced mRNA expression of *IL-8* (**Figure 2A**). In contrast, in JEG3 cells, *IL-1β* and *IL-8* mRNA levels were undetectable in all groups (not shown); additionally, no rh-S-protein concentration altered basal *IL-6* levels (**Figure 2B**). In PMVECs, *IL-1β* and *IL-6* levels were significantly elevated by rh-S-protein again with a clear dose response evident, while only highest concentration of rh-S-protein significantly induced *IL-8* levels (**Figure 2C**), similar to the pattern seen with HTR8. However, in HEECs, *IL-1β* levels increases did not attain statistical significance; while *IL-6* levels were significantly induced by 100 and 1000ng/ml of rh-S-protein, and *IL-8* levels were only elevated by the highest concentration of rh-S-protein (**Figure 2D**). Similarly, ELISA analysis revealed significantly higher levels of IL-6 and IL-8 secretion in culture supernatants of HTR8 (**Figure 2E**) and PMVECs (**Figure 2F**) treated with 1000 ng/ml rh-S-protein *vs*. control, validating S-protein mediated increase in IL-6 and IL-8 transcription in HTR8 and PMVECs. However, 10 ng/ml rh-S-protein treatment did not induce secretion levels of either cytokine in these cell types. These findings indicate: 1) low concentrations of S-protein appear sufficient to induce of *IL-1β* and *IL-6* levels, but a higher concentration is required to induce *IL-8* levels in HTR8, PMVECs and HEECs; 2) only higher concentration of rh-S-protein induces secretion of IL-6 and IL-8 levels in HTR8 and PMVECs; and 3) there is a clear inflammatory response to COVID-19 in vascular endothelial cells, potentially contributing to viral pathogenesis in pregnant women.

### Enhanced Chemokine Expression by rh-S-Protein in Trophoblast and Endothelial Cells

**Figure 3:**
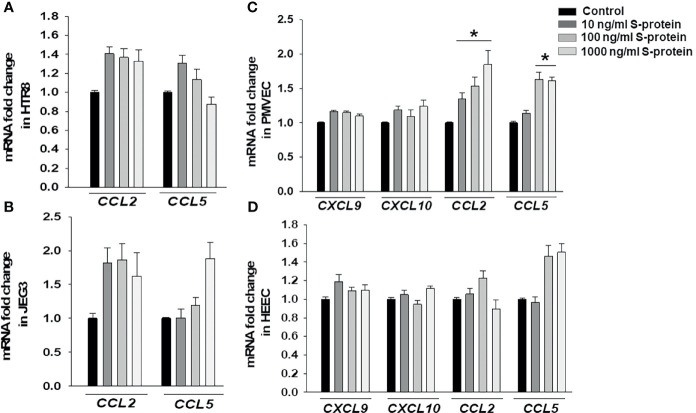
Expression chemokines mRNA in trophoblastic and endothelial cells cultures treated by rh-S-protein. qPCR analysis revealed to measure of expression levels of *CCL2, CCL5, CXCL9 and CXCL10* in HTR8/SV^Neo^ **(A)**, JEG3 **(B)**, PMVEC **(C)** and HEEC **(D)** cultures treated with 10, 100, or 1000 ng/ml rh-S-protein *vs.* control. Bars represent mean ± SEM, n=4; * *P<0.05 vs.* control.

Following confirmation that rh-S-protein treatment resulted in an alteration of pro-inflammatory gene expression, we compared mRNA expression levels of chemokines *CXCL9*, *CXCL10*, *CCL2*, and *CCL5* in HTR8, JEG3, PMVECs, and HEECs cultures. The qPCR analysis revealed that compared to mock-treated cells, levels of *CCL2* and *CCL5* in both HTR8 (**Figure 3A**) and JEG3 cells (**Figure 3B**) were not altered by any rh-S-protein exposure. Moreover, levels of *CXCL9* and *CXCL10* were undetectable and not induced by any rh-S-protein concentration in both HTR8 and JEG3 cells. In contrast, in PMVECs cultures, *CCL2* levels were significantly induced by all rh-S-protein concentrations in a dose-response fashion, while *CCL5* levels were only significantly increased at concentrations of rh-S-protein of 100 or 1000 ng/ml and whereas basal *CXCL9* and *CXCL10* levels were unchanged (**Figure 3C**). Again, in contrast to PMVECs, rh-S-protein did not affect expression of these cytokines in HEECs (**Figure 3D**).

### Elevated Tissue Factor (*F3*) Expression by rh-S-protein in Endothelial Cells, but Not Trophoblasts

**Figure 4:**
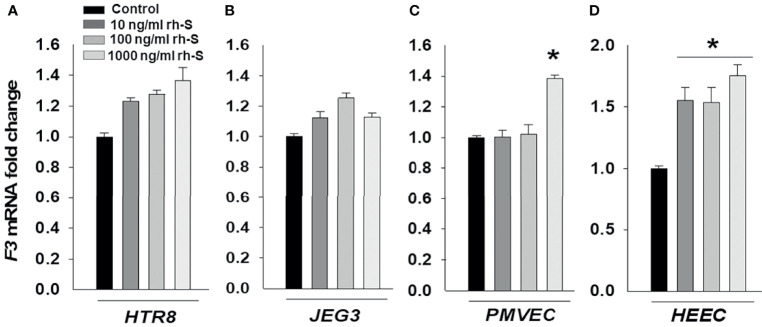
Increased tissue factor mRNA levels by rh-S-protein in endothelial cell, but not in trophoblast cell cultures. qPCR analysis measured tissue factor **(***F3)* mRNA expression in trophoblast cell lines HTR8/SV^Neo^ **(A)** and JEG3 **(B)** as well as endothelial PMVEC **(C)** and HEEC **(D)** cultures treated with mock-treated control or 10, 100, or 1000 ng/ml rh-S-protein. Bars represent mean ± SEM, n=3; * *P<0.05 vs.* control, 10 or 100 ng/ml rh-S-protein in PMVECs; and * *P<0.05 vs.* control in HEECs.

Thrombotic complications are frequent in COVID-19 patients and are associated with disease severity and mortality (29). F3 that is the primary initiator of coagulation is not normally expressed by endothelial cells or trophoblast, though its expression can be induced by proinflammatory cytokines. Thus, we evaluated expression levels of *F3* in cells treated with 10, 100, and 1000 ng/ml rh-S-protein to explore potential etiologies of placental thrombosis in SARS-CoV-2 infected pregnant women. After 24 hours treatment, qPCR results displayed no significant difference in mRNA expression levels of *F3* in either HTR8 (**Figure 4A**) or JEG3 (**Figure 4B**). However, 1000 ng/ml of rh-S-protein significantly increased *F3* levels in PMVECs (**Figure 4C**), whereas all concentrations induced *F3* levels in HEECs compared to control groups (**Figure 4D**).

### IFNγ Treatment in Combination With rh-S-Protein Augments Expression of Pro-Inflammatory Cytokines and Chemokines

**Figure 5:**
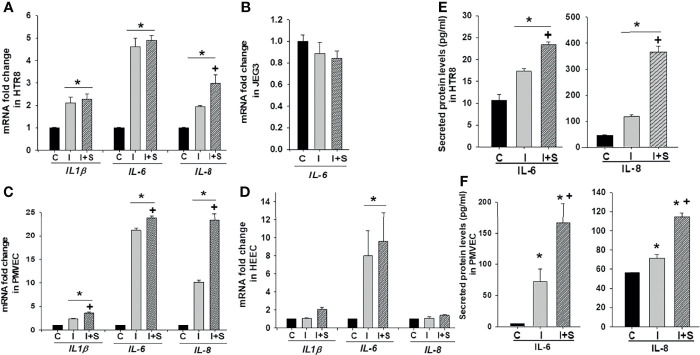
IFNγ with or without S-protein significantly increases expression of pro-inflammatory cytokines in trophoblastic and endothelial cell cultures. Expression of pro-inflammatory cytokines genes *IL-1β*, *IL-6*, *IL-8* was measured in HTR8/SV^neo^ **(A)**, JEG3 **(B)**, PMVECs **(C)**, and HEECs **(D)** treated with either control or 10 ng/ml rh-IFNγ ± 10 ng/ml rh-S-protein by qPCR. Bars represent mean ± SEM, n=3; * *P<0.05 vs.* control and + *P<0.05 vs.* rh-IFNγ alone. ELISA results revealed to measure secreted IL-6 and IL-8 protein levels in HTR8 **(E)** and PMVECs **(F)** by treatment with 10 ng/ml rh-IFNγ or rh-IFNγ + 10 ng/ml rh-S-protein. Bars represent mean ± SEM, n=3; *P<0.05 vs. control. + *P<0.05 vs*. IFNγ alone. C, Control; I, rh-IFNγ; and I+S, rh-IFNγ + rh-S-protein.

To explore whether the immunological state of pregnancy can promote adverse pregnancy outcomes in SARS-CoV-2 infected pregnant women, HTR8, JEG3, PMVECs and HEECs cultures were treated with 10 ng/ml rh-IFNγ ± 10 ng/ml rh-S-protein since a significant positive correlation was reported between IFNγ levels and disease severity in pregnant women (30). The expression of pro-inflammatory cytokines and chemokines levels were then measured. The qPCR analysis revealed that compared to mock-treated controls: 1) in HTR8 cells, rh-IFNγ alone significantly increased mRNA levels of the pro-inflammatory cytokines *IL-1β*, *IL-6*, *IL-8*. However, the combination of rh-IFNγ with rh-S-protein further elevated *IL-8* levels, but not *IL-1β* or *IL-6* levels (**Figure 5A**); 2) in JEG3 cells, rh-IFNγ alone or in combination with rh-S-protein did not alter *IL-6* levels, whereas *IL-1β* and *IL-8* levels were undetectable (**Figure 5B**); 3) in PMEVCs, rh-IFNγ alone significantly increased *IL-1β*, *IL-6* and *IL-8* levels, which are further induced by the combination of rh-IFNγ and rh-S-protein (**Figure 5C**); and 4) rh-IFNγ alone enhanced *IL-6* levels, and addition of rh-S-protein did not further induce cytokine mRNA levels in HEECs (**Figure 5D**). Further analysis by ELISA revealed that HTR8 (**Figure 5E**) and PMVEC cultures (**Figure 5F**) treated with 10 ng/ml IFNΥ displayed significantly higher IL-6 and IL-8 secretion levels, which are the further increased by the addition of rh-S-protein (**Figures 5E, F**).

**Figure 6:**
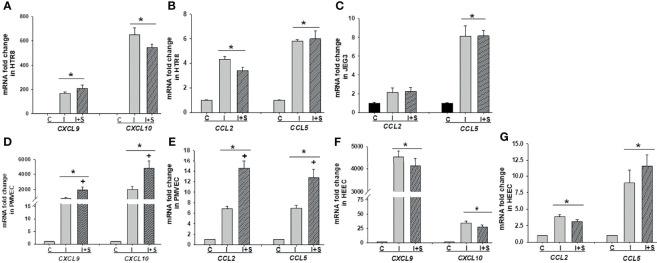
Recombinant IFNγ with or without S-protein significantly increases mRNA levels of chemokines in trophoblastic and endothelial cell cultures. Expression of chemokines *CXCL9*, *CXCL10* **(A)**; *CCL2 and CCL5* **(B)** in HTR8/SV^neo^, expression of chemokines *CCL2 and CCL5* **(C)** in JEG3*;* expression of chemokines *CXCL9*, *CXCL10* **(D)**, *CCL2 and CCL5* **(E)** in PMVECs; expression of chemokines *CXCL9*, *CXCL10* **(F)**, *CCL2 and CCL5* **(G)** in HEECs treated with either control or 10 ng/ml rh-IFNγ ± 10 ng/ml rh-S-protein treated cells. Bars represent mean ± SEM, n=3; *P<0.05 vs. control. + *P<0.05 vs*. IFNγ alone. C, Control; I, rh-IFNγ; and I+S, rh-IFNγ + rh-S-protein.

Following the same protocol with **Figure 5**, we evaluated the impact of rh-IFNγ on chemokine expression in these cells by qPCR and noted that mRNA levels of *CXCL9*, *CXCL10* (**Figure 6A**), and *CCL2* and *CCL5* (**Figure 6B**) were significantly enhanced by rh-IFNγ, but not further altered by addition of rh-S-protein in HTR8. In JEG3 cells, only *CCL5* levels were induced by rh-IFNγ, but again not further increased by adding rh-S-protein, whereas *CCL2* levels did not attain significance in any incubation condition (**Figure 6C**). Interestingly, in JEG3 cells, *CXCL9* and *CXCL10* levels were undetectable in both the control and rh-IFNγ treatment groups. Finally, rh-IFNγ significantly induced expression of *CXCL9*, *CXCL10* (**Figures 6D, F**), and *CCL2* and *CCL5* (**Figures 6E, G**) in both PMVECs and HEECs, and rh-S-protein further elevated their expression in PMVECs, but not HEECs (**Figures 6D–G**).

### Elevated *F3* Expression by rh-IFNγ in Combination With rh-S-Protein in Only PMVEC Cultures

**Figure 7:**
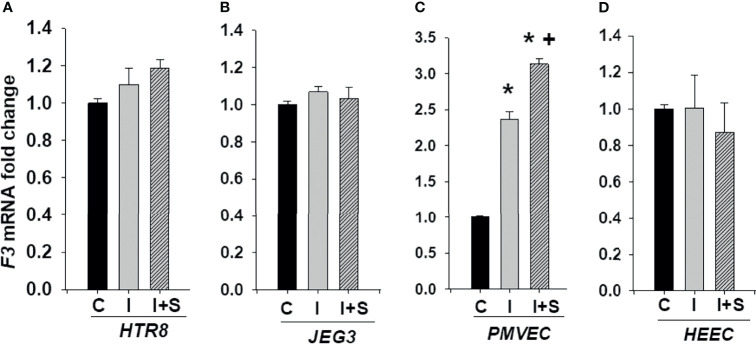
IFNγ with or without S-protein significantly increases tissue factor (*F3)* levels in only PMVECs. Expression of *F3* was measured in HTR8/SV^neo^ **(A)**, JEG3 **(B)**, PMVECs **(C)**, and HEECs **(D)** treated with either control or 10 ng/ml rh-IFNγ alone or in combination with 10 ng/ml rh-S-protein. Bars represent mean ± SEM, n=4 * *P<0.05 vs.* control. + *P<0.05 vs*. rh-IFNγ alone. C, Control; I, rh-IFNγ; and I+S, rh-IFNγ + rh-S-protein.

We next investigated whether enhanced rh-IFNγ contributes to the risk of thrombosis by measuring *F3* expression in trophoblastic HTR8 and JEG3 as well as endothelial PMVECs and HEECs cultures. **Figure 7** shows that *F3* mRNA levels are not induced by either rh-IFNγ alone or in combination with rh-S-protein in both HTR8 (**Figure 7A**) and JEG3 cells (**Figure 7B**). However, compared to control, *F3* mRNA levels were significantly higher in PMVECs treated with 10ng/ml rh-IFNγ and the combination of rh-IFNγ +10 ng/ml rh-S-protein further increased *F3* expression in PMVECs (**Figure 7C**). Interestingly, *F3* expression was not induced by either rh-IFNγ alone or in combination with rh-S-protein in HEECs, suggesting that interferon blocked spike protein induction of tissue factor (**Figure 7D**).

### Detection of SARS-CoV-2 Spike Protein Expression in Placenta

**Figure 8:**
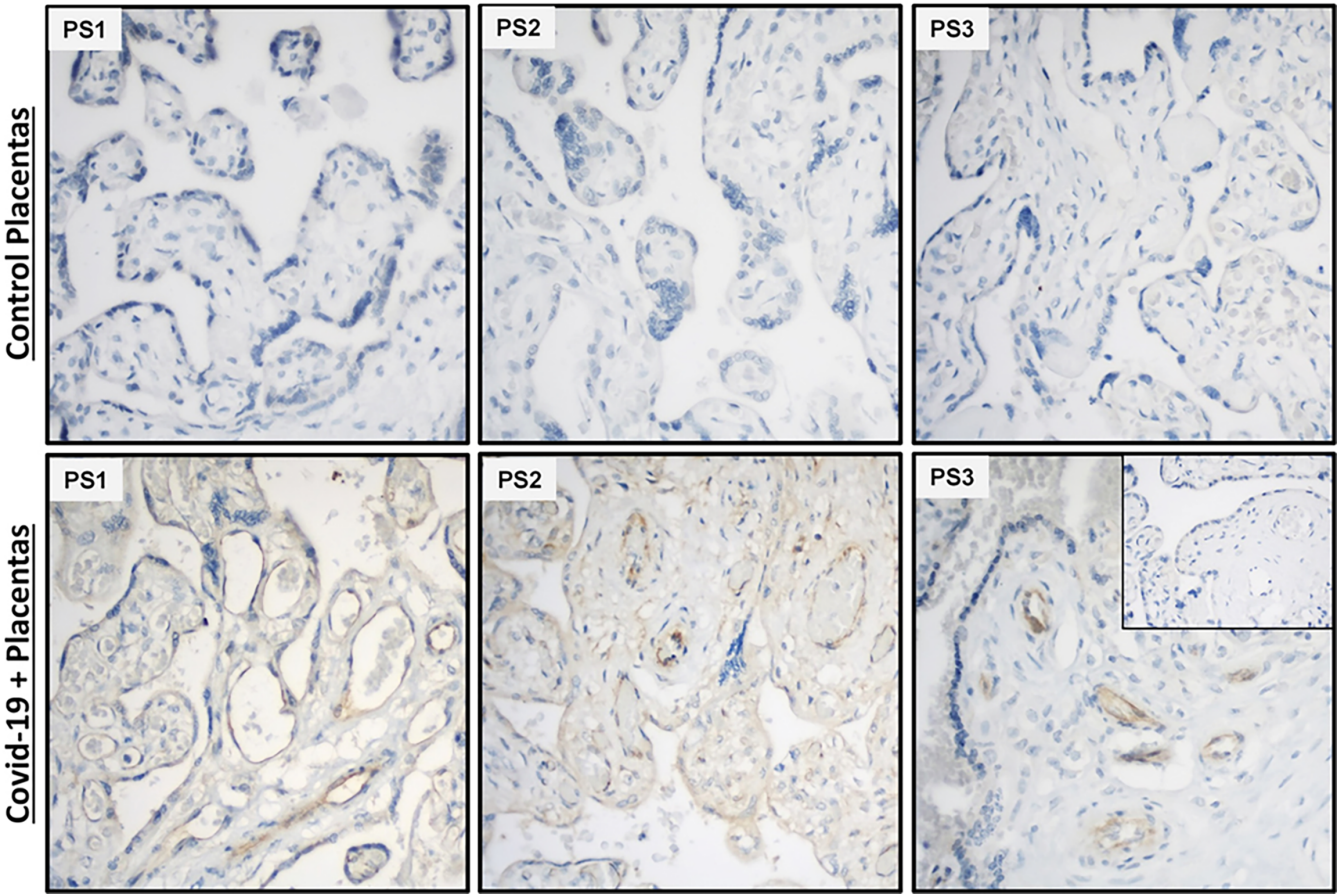
SARS-CoV-2 spike protein immunostaining in placental villi from women tested positive for Covid-19. Endothelial cells and trophoblastic layers display strong to weak SARS-CoV-2 spike protein immunoreactivity in placental sections from COVID-19 infected mothers (n=3), but not in cells in placental sections from non-infected mothers (n=3). Inset picture represents negative control incubated with non-immune IgG_2a_ at the same monoclonal IgG_2a_ primary antibody concentration. PS, Placental specimen; Original magnification: 40X.

Analysis of placental sections immunostained with anti-SARS-CoV-2 spike RBD revealed that endothelial cells as well as trophoblast layer displayed immunoreactivity in placental villi obtained from mothers who tested COVID-19 positive in the third trimester (**Figure 8**), whereas no reaction was detected in either endothelial cells or other cells in the placental villi obtained from gestational age-matched normal pregnancies (**Figure 8**).

## Discussion

Viral infections are a major cause of global morbidity and mortality. During pregnancy, viral infections that breach the placental barrier are frequently responsible for devastating effects on fetal development and maternal health (31). Pregnant women are more susceptible to several viral infections related to pregnancy-specific immune adaptation, which promotes maternal tolerance of the semi-allogenic fetus and enables viral infections (20, 32). Therefore, pregnant women represent a vulnerable population that has been carefully monitored during the COVID-19 pandemic. Several studies have reported that compared to non-pregnant reproductive age women, COVID-19 infected pregnant women are at higher risk for: 1) severe illness; and 2) preterm birth and cesarean delivery as well as other adverse pregnancy outcomes (12, 13, 18). Therefore, this study evaluated the underlying mechanism(s) associated with the placental pathology observed in these women (33) to help explain both disease severity and adverse human pregnancy outcomes.

The presence of COVID-19 infection in pregnant patients raises the question concerning vertical transmission of SARS-CoV-2 to the fetus (21). Although rare, vertical transmission of SARS-CoV-2 has been reported with detectable SARS-CoV-2 in the placenta, predominantly in syncytiotrophoblasts (22, 34, 35). These findings indicate the importance of identifying cellular tropisms for SARS-CoV-2 at the maternal-fetal interface. Therefore, we first examined the cell specific expression of the documented viral entry receptors, *ACE2* and *TMPRSS2*, in primary cultured cells from the maternal-fetal interface. Our results reveal high expression of *ACE2* and *TMPRSS2* in syncytiotrophoblasts, and cytotrophoblasts compared to maternal first and third trimester decidual cells, consistent with the pathological detection of SARS-CoV-2 viral RNA in the placenta. Surprisingly, these expression profile for SARS-CoV-2 viral entry receptors are the opposite of those found with Zika virus infection since Zika virus viral entry molecules are highly expressed in maternal decidual cells compared to trophoblast (25), indicating the importance of confirming individual virus-specific mechanisms in the placenta. Previous studies demonstrated ACE2 expression in syncytiotrophoblasts, cytotrophoblasts, endothelial and vascular smooth muscle cells in placenta villi (36, 37). These data were further supported by recent studies using single cell RNA-sequence analysis (15, 38). However, co-expression of both entry molecules was observed only in the chrorioamnionic membranes, whereas TMPRSS2 levels were not detected in several placental cells (38). Similarly, we detected *ACE2*, but not *TMPRSS2* expression in decidua cells, PMVECs and HESCs, suggesting the likelihood that SARS-CoV-2 could infect placental cells by using alternative host entry molecule(s) recently identified by Gordon et al. (39). Thus, further studies are required to identify other molecules that play a role in host infection, *for example*, cathepsin L and furin are other candidate proteases that prime the S-protein of SARS-CoV-2 (40).

An excessive inflammatory response to SARS-CoV-2 is a major cause of disease severity as well as mortality in COVID-19 patients and is associated with high levels of circulating cytokines *i.e.*, IL-1β, IL-6, IL-7, IFNγ and TNFα and chemokines *i.e.*, CCL2, CLL3 and CXCL10 (1, 41, 42). Maternal infection and inflammation associated with COVID-19 could prompt potential pregnancy complications through this “cytokine storm”. Several studies reported increased expression of inflammatory biomarkers in pregnant women with COVID-19 (21, 30, 43). Thus, we assessed the impact of SARS-CoV-2 infection during pregnancy on placental inflammation as a potential cause of adverse pregnancy outcomes. We found that rh-S-protein treatment triggers increase of pro-inflammatory cytokines *IL-1β*, *IL-6*, *IL-8* and chemokines *CXCL9*, *CXCL10*, *CCL2*, *CCL5* in a cell-type specific manner. The increase levels of these inflammatory markers could exaggerate the fetal and maternal immune system that is associated with stillbirth, fetal growth restriction, preeclampsia and/or PTB. COVID-19 has been linked to an increase occurrence of preeclampsia (44) as well as a preeclamptic-like illness (45). Preeclampsia is also associated with increased IL-6 levels (46). Interestingly, we found elevated *IL-6* levels in HTR8 and endothelial cells, suggesting a potential similar pathogenesis.

Also, we previously reported that IL-1β and IL-6 are responsible for chorioamnionitis-associated PTB and weakened fetal membrane through intense generation of extracellular matrix degrading metalloproteases (26, 47, 48). In addition, IL-1β is a potent inhibitor of decidual cell progesterone receptor expression, which accompanies chorioamnionitis (48). Therefore, the higher pro-inflammatory cytokine and chemokine responses observed in SARS-CoV-2 infected pregnant women may help explain the association between SARS-CoV-2 infection and inflammation-associated PTB. COVID-19 infected pregnant women are potentially at increased risk of developing coagulopathy and/or thromboembolic complications since pregnancy represents a physiological pro-thrombotic stage (49). A recent study found that the placentas from women infected with COVID-19 displayed a type of injury associated with uteroplacental vascular insufficiency which has been associated with stillbirth, fetal growth restriction, preeclampsia, abruption and preterm birth (50). Therefore, we investigated *F3* levels in rh-S-protein treated endothelial and trophoblast cell cultures and found rh-S-protein induced *F3* levels in only endothelial PMVEC and HEEC cells.

F3 initiates the coagulation process by binding to activated factor VII to activate factor IX and X, and subsequently generate thrombin, which activates endothelial cells, platelets, leukocytes and propagates microvascular thrombosis (51). Our previous studies reported decidual cells generate tissue factor during decidualization contributing uterine and placental hemostasis (52, 53). Decidual hemorrhage induces significant thrombin from decidual cell tissue factor accounting for the associated consumptive coagulopathy as well as the link between abruption and development of preterm premature rupture of membranes and spontaneous preterm birth. Thrombin promotes the production of decidual cell-derived pro-inflammatory cytokines, and matrix-degrading metalloproteinases (54, 55) and inhibits decidual cell progesterone receptor expression by activation of the ERK1/2 pathway (56). Thus, these findings provide clear evidence of potential molecular mechanisms to account for the observed histopathological changes in the placenta from women infected with COVID-19.

IFNγ, a pleiotropic lymphokine, exerts important regulatory effects on many cell types, and is essential for the initiation of uterine vascular modifications, directly and through the recruitment of Natural Killer (NK) cells and maintenance of decidual integrity (57). Conversely, excess decidual IFNγ expression may inhibit uterine NK cell migration (58). Recently, Tanacan et al., reported significantly higher levels of IFNγ in COVID-19 infected pregnant women, most prominently in the third trimester samples (30). Therefore, we investigated the impact of rh-IFNγ on SARS-CoV-2 placental pathogenesis and observed that rh-IFNγ treatment significantly increased mRNA levels of pro-inflammatory cytokines *IL-1β*, *IL-6*, *IL-8*, chemokines *CXCL9*, *CXCL10, CCL2, CCL5* and *F3* in both trophoblast and endothelial cell lines. Moreover, rh-IFNγ in combination with rh-S-protein further induces the expression of pro-inflammatory cytokines, chemokines and *F3* in placental endothelial cells PMVECs, but not in HEECs isolated from the endometrium of non-pregnant women. Combination of results showing *in vitro* and *in situ* detection of SARS-Co-V-2 spike protein expression in endothelial cells in placental villi suggest that IFNγ and S-protein synergistically induce inflammation and vascular thrombosis specifically in placenta endothelial cells, which likely play an important role in linking COVID-19 infections with adverse pregnancy outcomes.

In conclusion, our results revealed that in placental cells, SARS-CoV-2 S-protein induces release of pro-inflammatory cytokines and chemokines, which likely contributes to the “cytokine storm” in pregnant women and potential cause of placental dysfunction as well as elevated F3 levels that may trigger the vascular thrombosis seen in the placentas of women infected with COVID-19. These findings also support the concept that SARS-CoV-2 infection in the presence of enhanced IFNγ levels amplifies pro-inflammatory cytokine release from placenta to cause utero-placental and/or feto-placental endothelial dysfunction, contributing to SARS-CoV-2-associated adverse pregnancy outcomes such as PTB, abruption, still birth, fetal growth restriction, and/or preeclampsia.

## Data Availability Statement

The raw data supporting the conclusions of this article will be made available by the authors, without undue reservation.

## Ethics Statement

The studies involving human participants were reviewed and approved by University of South Florida. The patients/participants provided their written informed consent to participate in this study.

## Author Contributions

OG-K, UK, FS, and CL designed research studies. OG-K, UK, and XG discussed results and wrote the manuscript. OG-K and UK supervised experiments, contributed to data analyses. XG, NS, and VA performed experiments and contributed to data analyses. TS supplied placental specimen paraffin blocks. All authors performed critical evaluation of final version of results and agreed to submit manuscript for publication.

## Conflict of Interest

The authors declare that the research was conducted in the absence of any commercial or financial relationships that could be construed as a potential conflict of interest.

## Publisher’s Note

All claims expressed in this article are solely those of the authors and do not necessarily represent those of their affiliated organizations, or those of the publisher, the editors and the reviewers. Any product that may be evaluated in this article, or claim that may be made by its manufacturer, is not guaranteed or endorsed by the publisher.

## References

[1] Huang C Wang Y Li X Ren L Zhao J Hu Y. Clinical Features of Patients Infected With 2019 Novel Coronavirus in Wuhan, China. Lancet (2020) 395(10223):497–506. doi: 10.1016/S0140-6736(20)30183-5 31986264 PMC7159299

[2] Tahir Ul Qamar M. Structural Basis of SARS-CoV-2 3CL(Pro) and Anti-COVID-19 Drug Discovery From Medicinal Plants. J Pharm Anal (2020) 10(4):313–9. doi: 10.1016/j.jpha.2020.03.009 PMC7156227 32296570

[3] Abolnezhadian F Makvandi M Alavi SM Azaran A Jalilian S Rashno M. Prevalence of SARS-CoV-2 in Patients With Severe Pneumonia in Khuzestan Province, Iran. Iran J Allergy Asthma Immunol (2020) 19(5):471–7. doi: 10.18502/ijaai.v19i5.4462 33463114

[4] Aboudounya MM Heads RJ. COVID-19 and Toll-Like Receptor 4 (TLR4): SARS-CoV-2 May Bind and Activate TLR4 to Increase ACE2 Expression, Facilitating Entry and Causing Hyperinflammation. Mediators Inflamm (2021) 2021:8874339. doi: 10.1155/2021/8874339 33505220 PMC7811571

[5] Angoulvant F Ouldali N Yang DD Filser M Gajdos V Rybak A. Coronavirus Disease 2019 Pandemic: Impact Caused by School Closure and National Lockdown on Pediatric Visits and Admissions for Viral and Nonviral Infections-A Time Series Analysis. Clin Infect Dis (2021) 72(2):319–22. doi: 10.1093/cid/ciaa710 PMC7314162 33501967

[6] Zhu N Zhang D Wang W Li X Yang B Song J. A Novel Coronavirus From Patients With Pneumonia in China, 2019. N Engl J Med (2020) 382(8):727–33. doi: 10.1056/NEJMoa2001017 PMC7092803 31978945

[7] Holshue ML DeBolt C Lindquist S Lofy KH Wiesman J Bruce H. First Case of 2019 Novel Coronavirus in the United States. N Engl J Med (2020) 382(10):929–36. doi: 10.1056/NEJMoa2001191 PMC7092802 32004427

[8] Chen N Zhou M Dong X Qu J Gong F Han Y. Epidemiological and Clinical Characteristics of 99 Cases of 2019 Novel Coronavirus Pneumonia in Wuhan, China: A Descriptive Study. Lancet (2020) 395(10223):507–13. doi: 10.1016/S0140-6736(20)30211-7 PMC7135076 32007143

[9] Walls AC Park YJ Tortorici MA Wall A McGuire AT Veesler D. Structure, Function, and Antigenicity of the SARS-CoV-2 Spike Glycoprotein. Cell (2020) 181(2):281–92.e6. doi: 10.1016/j.cell.2020.02.058 32155444 PMC7102599

[10] Rabi FA Al Zoubi MS Kasasbeh GA Salameh DM Al-Nasser AD. SARS-CoV-2 and Coronavirus Disease 2019: What We Know So Far. Pathogens (2020) 9(3):231–50. doi: 10.3390/pathogens9030231 PMC7157541 32245083

[11] V’Kovski P Kratzel A Steiner S Stalder G Thiel V. Coronavirus Biology and Replication: Implications for SARS-CoV-2. Nat Rev Microbiol (2021) 19(3):155–70. doi: 10.1038/s41579-020-00468-6 PMC7592455 33116300

[12] Zambrano LD Ellington S Strid P Galang RR Oduyebo T Tong VT. Update: Characteristics of Symptomatic Women of Reproductive Age With Laboratory-Confirmed SARS-CoV-2 Infection by Pregnancy Status - United States, January 22-October 3, 2020. MMWR Morb Mortal Wkly Rep (2020) 69(44):1641–7. doi: 10.15585/mmwr.mm6925a1 PMC7643892 33151921

[13] Allotey J Stallings E Bonet M Yap M Chatterjee S Kew T. Clinical Manifestations, Risk Factors, and Maternal and Perinatal Outcomes of Coronavirus Disease 2019 in Pregnancy: Living Systematic Review and Meta-Analysis. BMJ (2020) 370:m3320. doi: 10.1136/bmj.m3320 32873575 PMC7459193

[14] Martinez-Portilla RJ Sotiriadis A Chatzakis C Torres-Torres J Espino YSS Sandoval-Mandujano K. Pregnant Women With SARS-CoV-2 Infection are at Higher Risk of Death and Pneumonia: Propensity Score Matched Analysis of a Nationwide Prospective Cohort (COV19Mx). Ultrasound Obstet Gynecol (2021) 57(2):224–31. doi: 10.1002/uog.23575 33320401

[15] Ellington S Strid P Tong VT Woodworth K Galang RR Zambrano LD. Characteristics of Women of Reproductive Age With Laboratory-Confirmed SARS-CoV-2 Infection by Pregnancy Status - United States, January 22-June 7, 2020. MMWR Morb Mortal Wkly Rep (2020) 69(25):769–75. doi: 10.15585/mmwr.mm6925a1 PMC7316319 32584795

[16] Wei SQ Bilodeau-Bertrand M Liu S Auger N. The Impact of COVID-19 on Pregnancy Outcomes: A Systematic Review and Meta-Analysis. CMAJ (2021) 193(16):E540–8. doi: 10.1503/cmaj.202604 PMC8084555 33741725

[17] Juan J Gil MM Rong Z Zhang Y Yang H Poon LC. Effect of Coronavirus Disease 2019 (COVID-19) on Maternal, Perinatal and Neonatal Outcome: Systematic Review. Ultrasound Obstet Gynecol (2020) 56(1):15–27. doi: 10.1002/uog.22088 32430957 PMC7276742

[18] Elsaddig M Khalil A. Effects of the COVID Pandemic on Pregnancy Outcomes. Best Pract Res Clin Obstet Gynaecol (2021) 73:125–36. doi: 10.1016/j.bpobgyn.2021.03.004 PMC7969862 33832868

[19] Pelayo J Pugliese G Salacup G Quintero E Khalifeh A Jaspan D. Severe COVID-19 in Third Trimester Pregnancy: Multidisciplinary Approach. Case Rep Crit Care (2020) 2020:8889487. doi: 10.1155/2020/8889487 33083063 PMC7563040

[20] Mor G Aldo P Alvero AB. The Unique Immunological and Microbial Aspects of Pregnancy. Nat Rev Immunol (2017) 17(8):469–82. doi: 10.1038/nri.2017.64 28627518

[21] Kotlyar AM Grechukhina O Chen A Popkhadze S Grimshaw A Tal O. Vertical Transmission of Coronavirus Disease 2019: A Systematic Review and Meta-Analysis. Am J Obstet Gynecol (2021) 224(1):35–53.e3. doi: 10.1016/j.ajog.2020.07.049 32739398 PMC7392880

[22] Hosier H Farhadian SF Morotti RA Deshmukh U Lu-Culligan A Campbell KH. SARS-CoV-2 Infection of the Placenta. J Clin Invest (2020) 130(9):4947–53. doi: 10.1172/JCI139569 PMC7456249 32573498

[23] Huang X Jia L Qian Z Jia Y Chen X Xu X. Diversity in Human Placental Microvascular Endothelial Cells and Macrovascular Endothelial Cells. Cytokine (2018) 111:287–94. doi: 10.1016/j.cyto.2018.09.009 30269024

[24] Schatz F Soderland C Hendricks-Munoz KD Gerrets RP Lockwood CJ. Human Endometrial Endothelial Cells: Isolation, Characterization, and Inflammatory-Mediated Expression of Tissue Factor and Type 1 Plasminogen Activator Inhibitor. Biol Reprod (2000) 62(3):691–7. doi: 10.1095/biolreprod62.3.691 10684811

[25] Guzeloglu-Kayisli O Guo X Tang Z Semerci N Ozmen A Larsen K. Zika Virus-Infected Decidual Cells Elicit a Gestational Age-Dependent Innate Immune Response and Exaggerate Trophoblast Zika Permissiveness: Implication for Vertical Transmission. J Immunol (2020) 205(11):3083–94. doi: 10.4049/jimmunol.2000713 33139490

[26] Lockwood CJ Basar M Kayisli UA Guzeloglu-Kayisli O Murk W Wang J. Interferon-Gamma Protects First-Trimester Decidual Cells Against Aberrant Matrix Metalloproteinases 1, 3, and 9 Expression in Preeclampsia. Am J Pathol (2014) 184(9):2549–59. doi: 10.1016/j.ajpath.2014.05.025 PMC4188280 25065683

[27] Guzeloglu Kayisli O Kayisli UA Basar M Semerci N Schatz F Lockwood CJ. Progestins Upregulate FKBP51 Expression in Human Endometrial Stromal Cells to Induce Functional Progesterone and Glucocorticoid Withdrawal: Implications for Contraceptive- Associated Abnormal Uterine Bleeding. PloS One (2015) 10(10):e0137855. doi: 10.1371/journal.pone.0137855 26436918 PMC4593551

[28] Guzeloglu-Kayisli O Semerci N Guo X Larsen K Ozmen A Arlier S. Decidual Cell FKBP51-Progesterone Receptor Binding Mediates Maternal Stress-Induced Preterm Birth. Proc Natl Acad Sci USA (2021) 118(11):e2010282118–30. doi: 10.1073/pnas.2010282118 PMC7980401 33836562

[29] Hanff TC Mohareb AM Giri J Cohen JB Chirinos JA. Thrombosis in COVID-19. Am J Hematol (2020) 95(12):1578–89. doi: 10.1002/ajh.25982 PMC7674272 32857878

[30] Tanacan A Yazihan N Erol SA Anuk AT Yucel Yetiskin FD Biriken D. The Impact of COVID-19 Infection on the Cytokine Profile of Pregnant Women: A Prospective Case-Control Study. Cytokine (2021) 140:155431. doi: 10.1016/j.cyto.2021.155431 33503581 PMC7810028

[31] Yockey LJ Lucas C Iwasaki A. Contributions of Maternal and Fetal Antiviral Immunity in Congenital Disease. Science (2020) 368(6491):608–12. doi: 10.1126/science.aaz1960 32381717

[32] Abu-Raya B Michalski C Sadarangani M Lavoie PM. Maternal Immunological Adaptation During Normal Pregnancy. Front Immunol (2020) 11:575197. doi: 10.3389/fimmu.2020.575197 33133091 PMC7579415

[33] Shanes ED Mithal LB Otero S Azad HA Miller ES Goldstein JA. Placental Pathology in COVID-19. Am J Clin Pathol (2020) 154(1):23–32. doi: 10.1093/ajcp/aqaa089 32441303 PMC7279066

[34] Pulinx B Kieffer D Michiels I Petermans S Strybol D Delvaux S. Vertical Transmission of SARS-CoV-2 Infection and Preterm Birth. Eur J Clin Microbiol Infect Dis (2020) 39(12):2441–5. doi: 10.1007/s10096-020-03964-y PMC7357443 32661809

[35] Hecht JL Quade B Deshpande V Mino-Kenudson M Ting DT Desai N. SARS-CoV-2 can Infect the Placenta and Is Not Associated With Specific Placental Histopathology: A Series of 19 Placentas From COVID-19-Positive Mothers. Mod Pathol (2020) 33(11):2092–103. doi: 10.1038/s41379-020-0639-4 PMC7395938 32741970

[36] Valdes G Neves LA Anton L Corthorn J Chacon C Germain AM. Distribution of Angiotensin-(1-7) and ACE2 in Human Placentas of Normal and Pathological Pregnancies. Placenta (2006) 27(2-3):200–7. doi: 10.1016/j.placenta.2005.02.015 16338465

[37] Levy A Yagil Y Bursztyn M Barkalifa R Scharf S Yagil C. ACE2 Expression and Activity are Enhanced During Pregnancy. Am J Physiol Regul Integr Comp Physiol (2008) 295(6):R1953–61. doi: 10.1152/ajpregu.90592.2008 18945956

[38] Pique-Regi R Romero R Tarca AL Luca F Xu Y Alazizi A. Does the Human Placenta Express the Canonical Cell Entry Mediators for SARS-CoV-2? Elife (2020) 9:e58716–3. doi: 10.7554/eLife.58716 PMC7367681 32662421

[39] Gordon DE Jang GM Bouhaddou M Xu J Obernier K White KM. A SARS-CoV-2 Protein Interaction Map Reveals Targets for Drug Repurposing. Nature (2020) 583(7816):459–68. doi: 10.1038/s41586-020-2286-9 PMC7431030 32353859

[40] Lukassen S Chua RL Trefzer T Kahn NC Schneider MA Muley T. SARS-CoV-2 Receptor ACE2 and TMPRSS2 are Primarily Expressed in Bronchial Transient Secretory Cells. EMBO J (2020) 39(10):e105114. doi: 10.15252/embj.20105114 32246845 PMC7232010

[41] Feng W Zong W Wang F Ju S. Severe Acute Respiratory Syndrome Coronavirus 2 (SARS-CoV-2): A Review. Mol Cancer (2020) 19(1):100. doi: 10.1186/s12943-020-01218-1 32487159 PMC7264920

[42] Tay MZ Poh CM Renia L MacAry PA Ng LFP. The Trinity of COVID-19: Immunity, Inflammation and Intervention. Nat Rev Immunol (2020) 20(6):363–74. doi: 10.1038/s41577-020-0311-8 PMC7187672 32346093

[43] Lombardi A Duiella S Li Piani L Comelli A Ceriotti F Oggioni M. Inflammatory Biomarkers in Pregnant Women with COVID-19: A Retrospective Cohort Study. Sci Rep (2021) 11(1):13350–7. doi: 10.1038/s41598-021-92885-7 PMC8233302 34172816

[44] Di Mascio D Khalil A Saccone G Rizzo G Buca D Liberati M. Outcome of Coronavirus Spectrum Infections (SARS, MERS, COVID-19) During Pregnancy: A Systematic Review and Meta-Analysis. Am J Obstet Gynecol MFM (2020) 2(2):100107. doi: 10.1016/j.ajogmf.2020.100107 32292902 PMC7104131

[45] Mendoza M Garcia-Ruiz I Maiz N Rodo C Garcia-Manau P Serrano B. Pre-Eclampsia-Like Syndrome Induced by Severe COVID-19: A Prospective Observational Study. BJOG (2020) 127(11):1374–80. doi: 10.1111/1471-0528.16339 PMC7300912 32479682

[46] Lockwood CJ Yen CF Basar M Kayisli UA Martel M Buhimschi I. Preeclampsia-Related Inflammatory Cytokines Regulate Interleukin-6 Expression in Human Decidual Cells. Am J Pathol (2008) 172(6):1571–9. doi: 10.2353/ajpath.2008.070629 PMC2408417 18467705

[47] Lockwood CJ Murk WK Kayisli UA Buchwalder LF Huang SJ Arcuri F. Regulation of Interleukin-6 Expression in Human Decidual Cells and its Potential Role in Chorioamnionitis. Am J Pathol (2010) 177(4):1755–64. doi: 10.2353/ajpath.2010.090781 PMC2947272 20724602

[48] Guzeloglu-Kayisli O Kayisli UA Semerci N Basar M Buchwalder LF Buhimschi CS. Mechanisms of Chorioamnionitis-Associated Preterm Birth: Interleukin-1beta Inhibits Progesterone Receptor Expression in Decidual Cells. J Pathol (2015) 237(4):423–34. doi: 10.1002/path.4589 26175191

[49] Servante J Swallow G Thornton JG Myers B Munireddy S Malinowski AK. Haemostatic and Thrombo-Embolic Complications in Pregnant Women With COVID-19: A Systematic Review and Critical Analysis. BMC Pregnancy Childbirth (2021) 21(1):108. doi: 10.1186/s12884-021-03568-0 33546624 PMC7863033

[50] Goldstein JA Miller ES Azad AH Otero S Mithal LB Shanes ED. Placental Pathology in COVID-19. Am J Clin Pathol (2020) 154(1):23–32. doi: 10.1093/ajcp/aqaa089 32441303 PMC7279066

[51] Canas CA Canas F Bautista-Vargas M Bonilla-Abadia F. Role of Tissue Factor in the Pathogenesis of COVID-19 and the Possible Ways to Inhibit it. Clin Appl Thromb Hemost (2021) 27:10760296211003983. doi: 10.1177/10760296211003983 33784877 PMC8020089

[52] Lockwood CJ Krikun G Papp C Toth-Pal E Markiewicz L Wang EY. The Role of Progestationally Regulated Stromal Cell Tissue Factor and Type-1 Plasminogen Activator Inhibitor (PAI-1) in Endometrial Hemostasis and Menstruation. Ann N Y Acad Sci (1994) 734:57–79. doi: 10.1111/j.1749-6632.1994.tb21736.x 7978955

[53] Lockwood CJ Nemerson Y Guller S Krikun G Alvarez M Hausknecht V. Progestational Regulation of Human Endometrial Stromal Cell Tissue Factor Expression During Decidualization. J Clin Endocrinol Metab (1993) 76(1):231–6. doi: 10.1210/jc.76.1.231 8421090

[54] Lockwood CJ Murk W Kayisli UA Buchwalder LF Huang ST Funai EF. Progestin and Thrombin Regulate Tissue Factor Expression in Human Term Decidual Cells. J Clin Endocrinol Metab (2009) 94(6):2164–70. doi: 10.1210/jc.2009-0065 PMC2690421 19276228

[55] Lockwood CJ Krikun G Caze R Rahman M Buchwalder LF Schatz F. Decidual Cell-Expressed Tissue Factor in Human Pregnancy and its Involvement in Hemostasis and Preeclampsia-Related Angiogenesis. Ann N Y Acad Sci (2008) 1127:67–72. doi: 10.1196/annals.1434.013 18443332

[56] Lockwood CJ Kayisli UA Stocco C Murk W Vatandaslar E Buchwalder LF. Abruption-Induced Preterm Delivery Is Associated With Thrombin-Mediated Functional Progesterone Withdrawal in Decidual Cells. Am J Pathol (2012) 181(6):2138–48. doi: 10.1016/j.ajpath.2012.08.036 PMC3509762 23058370

[57] Ashkar AA Di Santo JP Croy BA. Interferon Gamma Contributes to Initiation of Uterine Vascular Modification, Decidual Integrity, and Uterine Natural Killer Cell Maturation During Normal Murine Pregnancy. J Exp Med (2000) 192(2):259–70. doi: 10.1084/jem.192.2.259 PMC2193246 10899912

[58] Lockwood CJ Huang SJ Chen CP Huang Y Xu J Faramarzi S. Decidual Cell Regulation of Natural Killer Cell-Recruiting Chemokines: Implications for the Pathogenesis and Prediction of Preeclampsia. Am J Pathol (2013) 183(3):841–56. doi: 10.1016/j.ajpath.2013.05.029 PMC3763772 23973270

