# Supplementary material for: Regulation of Proinflammatory Molecules and Tissue Factor by SARS-CoV-2 Spike Protein in Human Placental Cells: Implications for SARS-CoV-2 Pathogenesis in Pregnant Women
Source: Front Immunol. 2022 Apr 7;13:876555. doi: 10.3389/fimmu.2022.876555 (PMC9022221; doi:10.3389/fimmu.2022.876555)
Supplement: Supplementary file 1 [file Table_1.docx]

| Gene name | Taqman ID number |
| --- | --- |
| *IL-1β*  *IL-6*  *IL-8*  *CCL-2*  *CCL-3*  *CCL-4*  *CCL-5*  *CXCL9*  *CXCL10*  *F3* | Hs01555410_m1  Hs00174131_m1  Hs00174103_m1  Hs00234140_m1  Hs00234142_m1  Hs99999148_m1  Hs00982282_m1  Hs00171065_m1  Hs00171042_m1  Hs01076029_m1 |

**Supplementary Table**

**S Table 1:** Gene name and probe ID used in this study
